# Validation of the Arabic version of the self-assessment of social cognitive impairments (ACSo) tool among a sample of patients with schizophrenia^☆^

**DOI:** 10.1016/j.scog.2025.100417

**Published:** 2026-01-06

**Authors:** Chadia Haddad, Samah Al Aswad, Hala Sacre, Francois Kazour, Helen Clery, Jérôme Graux, Sara Al Kadamani, Adella Ibrahim, Anthony Rizk, Pascale Salameh, Benjamin Calvet

**Affiliations:** a Inserm U1094, IRD UMR270, Univ. Limoges, CHU Limoges, EpiMaCT - Epidemiology of chronic diseases in tropical zone, Institute of Epidemiology and Global Health – Michel Dumas, OmegaHealth, Limoges, France; b Institut National de Santé Publique, d'Épidémiologie Clinique et de Toxicologie-Liban (INSPECT-LB), Beirut, Lebanon; c Research Department, Psychiatric Hospital of the Cross, Jal Eddib, Lebanon; d Faculty of Sciences, Lebanese University, Fanar, Lebanon; e Department of Psychiatry and Addictology, CHU Angers, Angers, France; f CHRU de Tours, Se Rétablir 37, Tours, France; g Department of Psychology and Social Sciences, Faculty of Arts and Sciences, Holy Spirit University of Kaslik (USEK), Jounieh, Mount Lebanon, Lebanon; h School of Medicine and Medical Sciences, Holy Spirit University of Kaslik, P.O. Box 446, Jounieh, Lebanon; i Faculty of Pharmacy, Lebanese University, Hadat, Lebanon; j School of Medicine, Lebanese American University, Byblos, Lebanon; k Department of Primary Care and Population Health, University of Nicosia Medical School, 2417, Nicosia, Cyprus; l Pôle Universitaire de Psychiatrie de l’Adulte et de la Personne Âgée, d’Addictologie, Centre Hospitalier Esquirol, Limoges, 87000, France; m Centre Hospitalier Esquirol, Unité de Recherche et d'Innovation, 87000, Limoges, France

**Keywords:** Social cognition, Neurocognition, Social cognitive complaint, Schizophrenia, Self-assessment scale

## Abstract

**Background:**

The social cognition aspect is today's research focus for improving the integration of patients with schizophrenia into society. This study analyzed the psychometric properties of the Arabic version of the Self-Assessment of Social Cognitive Impairments (ACSo) tool.

**Methods:**

A cross-sectional study at the Psychiatric Hospital of the Cross (HPC), Lebanon, enrolled 116 chronic inpatients between July and November 2023. Subjective assessment of social cognitive complaints was done using ACSo. Other clinical and objective measurements were collected to validate the ACSo tool.

**Results:**

ACSo factor analysis revealed a 4-factor solution using the Promax rotated matrix. The Cronbach's alpha value for the scale was 0.656. The ACSo total score positively correlated with its items and factors. In the entire patient population, the ACSo was positively correlated with cognitive complaints (*r* = 0.560; *p* < 0.0001), achieving concurrent validity. A significant negative correlation was found between facial emotion recognition (TREF) and the total ACSo scale (*r* = −0.246, *p* = 0.002). A significant negative correlation was found between the false belief theory of mind (TOM-15) and the total ACSo scale (*r* = −0.286, *p* = 0.001). Comprehension beliefs (TOM-15) were not associated with the total ACSo scale and subscales. A negative association was found between the empathy scale and the total ACSo scale.

**Conclusion:**

ACSo is a valuable tool for the self-assessment of social cognitive complaints in patients with schizophrenia since it demonstrated acceptable internal consistency and good concurrent and construct validity.

## 1 Background

Patients with schizophrenia show decreased performance in real-world social situations, which limits their ability to perceive and use social information effectively (Harvey and Penn, 2010). This reduced real-world functioning in individuals with schizophrenia seems to be related to impairments in various cognitive functions (Harvey and Penn, 2010). Thus, impaired social cognition mediates the relationship between cognition and social outcomes (Harvey and Penn, 2010). Extensive research has shown that individuals with schizophrenia exhibit deficits across all major domains of social cognition, including theory of mind, attributional style, social perception, and emotion processing (Javed and Charles, 2018). Longitudinal studies demonstrate that impairments in these four domains of social cognition stay stable over time, which makes them targets for intervention aiming to improve functional outcomes in patients with schizophrenia (Javed and Charles, 2018; McCleery et al., 2016).

Social cognition can be assessed using clinical and objective measures that target its specific elements; however, subjective tools may better capture individuals' lived experiences and can complement objective assessment. Developing an easily comprehensible self-assessment scale that is applicable across various psychiatric conditions may help clinicians identify therapeutic goals. Existing tools, including the Observable Social Cognition Rating Scale (OSCARS), the Cognitive Assessment Interview (CAI), and the Schizophrenia Cognition Rating Scale (SCoRS), vary in focus, format, and the domains they evaluate. The CAI is a semi-structured interview assessing general cognitive functioning across multiple domains, with limited attention to social cognition (Ventura et al., 2010). The SCoRS evaluates cognitive deficits and their impact on daily functioning and can serve as a self-report, informant-report, or clinician-rated scale (Keefe et al., 2015). Although the SCoRS has been translated into Arabic, it does not specifically assess patients' subjective awareness of social cognitive impairments. Until recently, subjective social cognition complaints were gathered mainly through clinical interviews, with the OSCARS representing the only structured option. Developed in 2015 by Healey and colleagues, the OSCARS is an 8-item self-report or informant-report measure assessing key domains such as theory of mind, attributional style, and emotion perception (Healey et al., 2015). The scale has demonstrated strong psychometric properties across multiple cultural contexts, including Arabic (Fekih-Romdhane et al., 2025), Spanish (Angela et al., 2021), Turkish (Özaslan and Yildiz, 2021), and Persian (Paliziyan et al., 2021) adaptations.

More recently, the ACSo (Self-Assessment of Social Cognition Impairments) was developed as a simple, transdiagnostic self-assessment tool to evaluate subjective complaints of social cognition among patients with schizophrenia. The scale was created as part of a collaborative effort made possible by the Institute of Psychiatry's GDR 3557. A preliminary study tested a first self-administered version comprising 20 items organized into four five-item groups (emotional processes, social perception and knowledge, theory of mind, and attributional biases) (Morlec, 2015). Psychometric evaluation of this initial version (personal data) led to a revised, shorter 12-item scale organized into four groups of three items (Graux et al., 2019). Unlike the OSCARS, which focuses on observable behaviors from clinician/informant perspectives, the ACSo measures patients' subjective awareness of their social cognitive difficulties across domains, including social perception, theory of mind, emotion perception, and attributional style. Validating the ACSo is particularly important because it captures patients' insight into their social cognition, an aspect strongly linked to engagement in psychosocial interventions and functional outcomes.

To date, no tools measuring subjective social cognitive complaints have been validated in Lebanon or other Arab countries. Translating and validating the ACSo into Arabic is essential to provide a culturally appropriate measure, given the central role of social cognition in functional recovery. A brief, reliable, and domain-specific tool could help clinicians identify patient-reported deficits and guide remediation strategies. Therefore, this study aimed to evaluate the psychometric properties of the Arabic version of the ACSo, originally developed and validated in French, to determine its utility among Lebanese patients with schizophrenia.

## 2 Methods

### 2.1 Study design and participants

A cross-sectional study was carried out on 116 chronic inpatients with schizophrenia disorders, diagnosed according to DSM-5 criteria, and recruited from the Psychiatric Hospital of the Cross (HPC), Lebanon. Diagnosis was established by a clinical psychiatrist based on a comprehensive clinical interview, documented in the medical file, and subsequently verified by the interviewer through chart review. The study was conducted between July and November 2023. Inclusion criteria were age 18–65 years, a minimum of five years of education, and clinical stability. Patients were excluded if they had an intellectual disability, mood disorders, a neurological history of substance abuse, or other comorbidities that could interfere with neuropsychological testing. Fifty healthy controls were recruited from HPC staff and matched to patients by age, education level, and sex. Controls were performing their work normally and reported not taking any psychiatric or other relevant medications; based on these criteria, they were considered healthy. No compensation was provided for participation.

### 2.2 Procedure

A total of 161 patients who met the inclusion criteria were initially selected. Of these, 38 were discharged before assessment, five refused to participate, and two were excluded due to inability to respond to questions, resulting in a final sample of 116 patients. For the control group, 78 healthy individuals were recruited from HPC. Of these, 23 left the hospital, and five refused to participate, yielding a final control of 50 participants. The data were collected through structured interviews with the participants and entered into SPSS by a single trained professional to minimize data access and protect participant confidentiality. All data entries were subsequently double-checked for accuracy by a second team member who conducted the statistical analyses.

### 2.3 Translation procedure

The ACSo scale was translated from French to Arabic using forward-backward translation methodology. Two healthcare professionals whose native language is Arabic independently translated the original scale into Arabic. Two different translators, who had not seen the original French version, then back-translated the Arabic version into French. The research team compared the back-translation with the original French text to identify any discrepancies in meaning or translation errors. Permission to translate and validate the ACSo scale was obtained from the primary authors through a signed research agreement (Graux et al., 2019).

### 2.4 Measures

The questionnaire was administered in Arabic, the native language in Lebanon. The first section collected sociodemographic and clinical data, including age, educational level, marital status, monthly income, schizophrenia subtype, family history of mental disorders, duration of hospitalization, duration of illness, and current treatment (molecule type and dose). The second section included the measures described below.

#### 2.4.1 Self-Assessment of Social Cognition Impairments (ACSo)

The ACSo is a 12-item self-report questionnaire designed to evaluate subjective social cognition complaints in individuals with serious mental illness or autism spectrum disorder (Graux et al., 2019). The scale assesses four domains with three items each: social perception and knowledge, emotional processes, theory of mind, and attributional biases. Participants rate each item on a 5-point Likert scale ranging from 0 (never) to 4 (very often), with higher total scores indicating greater subjective social cognitive impairment.

#### 2.4.2 The Self-assessment Scale of Cognitive Complaints in Schizophrenia (SASCCS)

The SASCCS is a 21-item self-report tool validated in Arabic that assesses patients' subjective cognitive impairment in schizophrenia (Johnson et al., 2009). It evaluates five cognitive domains: memory (6 items: 1–3, 9–11), praxia (5 items: 4–8), attention (5 items: 12–16), executive functions (3 items: 17–19), and language (2 items: 20–21). Items are rated on a 5-point Likert scale from 0 (never) to 4 (very frequently), with the total score calculated by summing all responses. This scale has been previously validated in the Lebanese population (Haddad et al., 2023). In the current study, Cronbach's alpha was 0.816.

#### 2.4.3 Questionnaire of Cognitive and Affective Empathy (QCAE)

The QCAE is a 31-item self-administered tool that assesses cognitive and affective empathy in adults (Reniers et al., 2009). Participants respond using a 4-point Likert scale ranging from strongly disagree to strongly agree. The scale comprises two subscales: cognitive empathy (19 items), assessing understanding of others' emotional states, and affective empathy (12 items), measuring the ability to vicariously experience others' emotions. The total empathy score is derived by summing both subscale scores. In the present study, Cronbach's alpha for the total scale was 0.829.

#### 2.4.4 Measures of Objective Social Cognition

##### 2.4.4.1 Facial Emotion Recognition Test (TREF)

The Facial Emotion Recognition Test (TREF) assesses the ability to recognize six basic emotions: joy, anger, sadness, fear, disgust, and contempt (Gaudelus et al., 2015). The test consists of 54 color photographs of six different models (three men and three women of varying ages). Each emotion is presented at nine intensity levels ranging from 20 % to 100 %, with each photograph displayed for 10 s. For this study, emotion labels were translated into Arabic and displayed on a phone screen, with responses recorded manually on a scoring sheet. Responses were scored dichotomously as correct or incorrect emotion identification. In the current study, Cronbach's alpha for the total scale was 0.830.

##### 2.4.4.2 Theory of Mind-15 (TOM-15)

The TOM-15 test is a false-belief test consisting of 15 stories: 8 first-order and 7 s-order theory of mind scenarios (Desgranges et al., 2012). Each story describes everyday situations that create a false belief in one of the characters. The test comprises two components using the same stories: a false belief task and a comprehension task, each with different questions. Stories were translated into Arabic, printed individually, and presented to participants according to standardized administration procedures, with responses recorded on a scoring sheet. Total scores for both the false belief task and comprehension task (each ranging from 0 to 15) were calculated by summing correct responses. In the current study, Cronbach's alpha was 0.812 for the false belief scale and 0.822 for the comprehension task.

#### 2.4.5 Clinical measures

##### 2.4.5.1 The Positive and Negative Syndrome Scale (PANSS)

The PANSS is a 30-item clinician-rated scale validated in Lebanon for assessing symptom severity in schizophrenia (Hallit et al., 2017). It comprises three subscales: positive symptoms (7 items: P1–P7), negative symptoms (7 items: N1–N7), and general psychopathology (16 items: G1–G16) (Hallit et al., 2017). Each item is rated from 1 (absent) to 7 (extremely severe), with subscale scores calculated by summing the respective items (Kay et al., 1987). In this study, Cronbach's alpha was 0.891.

##### 2.4.5.2 The Calgary Depression Scale for Schizophrenia (CDSS)

The CDSS is a 9-item clinician-rated scale specifically designed to assess depression in schizophrenia (Addington et al., 1993). Items evaluate depression, self-deprecation, hopelessness, guilt, pathological guilt, morning depression, early wakening, suicide, and observed depression. Each item is scored from 0 (absent) to 3 (severe). The total score (ranging from 0 to 27) indicates the severity of depressive symptoms, with higher scores suggesting greater depression. In this study, Cronbach's alpha was 0.831.

##### 2.4.5.3 The Insight Scale (IS) for psychosis

The IS is an 8-item self-report measure assessing insight in psychosis across three dimensions (Birchwood et al., 1994). The scale evaluates (1) awareness of illness (items 1 and 8), (2) awareness of symptoms (items 2 and 7), and (3) awareness of need for treatment (items 3, 4, 5, and 6, summed and divided by 2). Each subscale yields a score from 1 to 4, where scores of 3–4 indicate good insight and 1–2 reflect poor insight. In this study, Cronbach's alpha was 0.521.

### 2.5 Data analysis

All statistical analyses were conducted using SPSS version 25.0 (IBM Corp., Armonk, NY). Descriptive statistics were calculated, with categorical variables presented as frequencies and percentages and continuous variables as means and standard deviations. Normality of the ACSo scale was assessed through visual inspection of histograms and by verifying that skewness and kurtosis values fell within the acceptable range (|1.96|). Independent samples *t*-tests were used to compare continuous variables between groups, while chi-square tests were used for categorical variables.

Psychometric validation proceeded as follows: Exploratory Factor Analysis (EFA) with principal component analysis and Promax rotation was conducted to identify the factor structure of the Arabic ACSo. The Kaiser-Meyer-Olkin (KMO) measure of sampling adequacy and Bartlett's test of sphericity were examined to confirm suitability for factor analysis. Factors with eigenvalues >1.0 were retained. Internal consistency reliability was assessed using Cronbach's alpha, with values ≥0.90 indicating excellent reliability, ≥ 0.80 good reliability, ≥ 0.70 acceptable reliability, and < 0.60 questionable or poor reliability (Tavakol and Dennick, 2011).

Convergent and divergent validity were examined using Pearson correlation coefficients between ACSo total scores and clinical measures (PANSS, CDSS, IS), cognitive measures (SASCCS), empathy (QCAE), and objective social cognition measures (TREF, TOM-15).

Multivariate analyses were performed to identify predictors of ACSo scores. Linear regression analysis was conducted with the ACSo total score as the dependent variable. Multivariate analysis of covariance (MANCOVA) was performed with ACSo subscale scores as dependent variables. Variables demonstrating *p* < 0.20 in bivariate analyses were included in multivariate models to control for potential confounding. Statistical significance was set at *p* < 0.05 for all analyses.

## 3 Results

### 3.1 Sociodemographic characteristics

Sociodemographic characteristics of the participants are presented in Table 1. Patients with schizophrenia had a mean age of 52.53 ± 7.26 years, and 65.5 % were male. The majority were single (93.1 %) and had low monthly income (70.7 %), while half (50.0 %) had completed secondary education. Approximately one-third (35.3 %) reported a family history of psychiatric illness. Regarding clinical characteristics, 50.9 % had paranoid subtype schizophrenia, with mean illness duration of 25.19 ± 9.67 years and mean hospitalization duration of 16.03 ± 8.52 years.

**Table 1:** Sociodemographic and clinical characteristics of the total sample (*N* = 166).

	Schizophrenia patients (*N* = 116)	Healthy control (*N* = 50)	*p*-Value
	Frequency (%)	Frequency (%)	
Sex			
Male	76 (65.5 %)	26 (52.0 %)	0.101
Female	40 (34.5 %)	24 (48.0 %)	
Missing values	0	0	
Education level			
Complementary	41 (35.3 %)	11 (22.0 %)	0.228
Secondary	58 (50.0 %)	16 (32.0 %)	
University	17 (14.7 %)	7 (14.0 %)	
Missing values	0	16 (32.0 %)	
Marital status			
Single/widowed/divorced	108 (93.1 %)	7 (14.0 %)	<0.001
Married	8 (6.9 %)	27 (54.0 %)	
Missing values	0	16 (32.0 %)	
Monthly income			
No income	32 (27.6 %)	0 (0.0 %)	<0.001
<1000 $	50 (43.1 %)	25 (50.0 %)	
1000–2000 $	33 (28.4 %)	7 (14.0 %)	
>2000 $	1 (0.9 %)	2 (4.0 %)	
Missing values	0	16 (32.0 %)	
Family history of psychiatric illness			
Yes	41 (35.3 %)	4 (8.0 %)	0.005
No	73 (62.9 %)	30 (60.0 %)	
Missing values	2 (1.7 %)	16 (32.0 %)	
Diagnostic (DSM- V)			
Paranoid	59 (50.9 %)		
Disorganized	6 (5.2 %)		
Undifferentiated	13 (11.2 %)		
Schizoaffective	28 (24.1 %)		
Missing values	10 (8.6 %)		
	Mean ± SD	Mean ± SD	
Age	52.53 ± 7.26	51.74 ± 7.58	0.288
Duration of illness in years	25.19 ± 9.67		
Duration of hospitalization in years	16.03 ± 8.52		
PANSS total score	77.50 ± 4.85		
Positive	18.14 ± 9.58		
Negative	17.50 ± 8.06		
General psychopathology	41.84 ± 16.33		
Insight scale	5.17 ± 2.22		
Calgary scale	4.85 ± 4.75		

The healthy control group was matched to the schizophrenia group on age, sex, and education level (all *p* > 0.05). However, the groups differed significantly on marital status, monthly income, and family history of psychiatric illness (all *p* < 0.05).

### 3.2 Comparison of the quantitative scales between schizophrenia patients and healthy controls

The schizophrenia group scored significantly higher means than the healthy controls in social cognitive complaint tests (more complaints). Also, significantly higher mean cognitive complaints (SASCCS scale) were found among patients with schizophrenia compared with healthy controls. Considering the facial emotion recognition, theory of mind, and empathy scales, significantly lower mean scales were found among patients with schizophrenia compared with the healthy control group (Table 2).

**Table 2:** Comparison of the quantitative scales used between the schizophrenia patients and healthy control groups.

	Schizophrenia patients (N = 116)	Healthy control (N = 50)	p-Value
	Frequency (%)	Frequency (%)	
Self-assessment of social cognition impairments (ACSo)^a^			
Total score	12.78 ± 8.46	7.75 ± 4.80	**<0.001**
Factor 1: Social perception	3.48 ± 3.78	2.04 ± 1.74	**0.001**
Factor 2: Attributional bias	3.56 ± 3.75	2.94 ± 2.82	0.238
Factor 3: Emotional perception	2.85 ± 2.70	1.16 ± 1.40	**<0.001**
Factor 4: Theory of mind	2.92 ± 3.07	1.54 ± 2.12	**0.001**
Facial emotion recognition (TREF)	21.84 ± 6.64	28.34 ± 5.91	**<0.001**
Theory of Mind (TOM-15)			
False beliefs	8.45 ± 3.51	12.38 ± 2.51	**<0.001**
Comprehension	10.36 ± 3.48	13.51 ± 1.81	**<0.001**
Self-Assessment Scale of Cognitive Complaints in Schizophrenia (SASCCS)^a^	22.77 ± 14.69	13.28 ± 9.67	**<0.001**
Questionnaire of Cognitive and Affective Empathy (QCAE)	85.74 ± 17.22	92.92 ± 14.58	**0.012**
Cognitive empathy	52.25 ± 11.81	58.82 ± 9.44	**<0.001**
Affective empathy	33.48 ± 7.54	34.10 ± 7.56	0.634

a For both the ACSo and SASCCS scales, lower scores reflect fewer self-reported complaints related to social cognition and general cognition, respectively. Values in bold indicate statistical significance (p < 0.05).

### 3.3 Validity of internal structure

A factor analysis was run to test the construct validity of the ACSo scale using principal component analysis as the extraction method. All items of the ACSo scale could be extracted from the list, and the scale converged on a 4-factor solution using the Promax rotated matrix with an eigenvalue greater than 1, accounting for 57.49 % of the variance (Bartlett's sphericity test *p* < 0.001, KMO = 0.685) (Table 3). Cronbach's alpha for the scale was 0.656.

**Table 3:** Factor analysis of the ACSo scale among patients with schizophrenia.

Promax rotated matrix				
Item	Factor 1	Factor 2	Factor 3	Factor 4
Item 9	0.778			
Item 2	0.773			
Item 1	0.740			
Item 4		0.833		
Item 12		0.669		
Item 7		0.608		
Item 5		0.451		
Item 11			0.685	
Item 8			0.648	
Item 3				0.597
Item 6				0.500
Item 10				0.439
Percentage variance explained = 57.49 %	23.51	14.68	10.14	9.16
Kaiser-Meyer-Olkin (KMO)	0.685			
Bartlett's test of sphericity	<0.001			
Cronbach alpha = 0.656	0.694	0.533	0.421	0.446

Factor 1: Social perception; Factor 2: Attributional bias; Factor 3: emotional perception; Factor: Theory of mind.

A Cronbach alpha values <0.60 are considered questionable or poor.

The ACSo demonstrated adequate internal structure, with total scores positively correlating with all items (*r* = 0.251–0.623) and subscales (*r* = 0.506–0.716). Item-subscale correlations showed variable patterns, with some items correlating more strongly with their designated subscale than others (Supplementary Table 1).

### 3.4 Convergent validity

The ACSo total score was positively correlated with the SASCCS total score (r = 0.560, *p* < 0.001), achieving concurrent validity.

For facial emotion recognition (TREF), negative correlations were found with ACSo total score (*r* = −0.246, *p* = 0.002), social perception subscale (*r* = −0.182, *p* = 0.022), and theory of mind subscale (*r* = −0.270, *p* = 0.001).

For theory of mind, the TOM-15 false-belief test correlated negatively with the ACSo total score (*r* = −0.286, p = 0.001) and the social perception subscale (*r* = −0.230, *p* = 0.036). The TOM-15 comprehension task showed no significant associations with any ACSo scores.

The PANSS total score and general psychopathology subscale were positively correlated with the ACSo total score and the attributional bias subscale (Table 4). Negative correlations were found between QCAE scores (total, affective, and cognitive empathy) and ACSo total score and all subscales except attributional bias.

**Table 4:** Pearson correlations between ACSo social cognitive complaints and associated factors in the total sample.

	Total ACSo score	Factor 1: Social perception	Factor 2: Attributional bias	Factor 3: emotional perception	Factor 4: Theory of mind
	Pearson correlation coefficients (r)	Pearson correlation coefficients (r)	Pearson correlation coefficients (r)	Pearson correlation coefficients (r)	Pearson correlation coefficients (r)
**Facial emotion recognition** **(TREF)**	−0.246	−0.182	−0.052	−0.147	−0.270
*p-value*	**0.002**	**0.022**	0.515	0.065	**0.001**
**Theory of Mind (TOM-15)**					
False beliefs	−0.286	−0.230	−0.213	0.078	0.022
*p-value*	**0.001**	**0.036**	0.054	0.486	0.843
Comprehension	−0.155	−0.051	−0.016	0.100	0.097
*p-value*	0.087	0.669	0.894	0.395	0.411
**Self-Assessment Scale of Cognitive Complaints in Schizophrenia (SASCCS)**	0.560	0.486	0.275	0.404	0.276
*p-value*	**<0.001**	**<0.001**	**<0.001**	**<0.001**	**<0.001**
**Questionnaire of Cognitive and** **Affective Empathy (QCAE)**	−0.413	−0.273	−0.093	−0.336	−0.246
*p-value*	**<0.001**	**0.005**	0.348	**0.001**	**0.012**
Cognitive empathy	−0.447	−0.302	−0.153	−0.300	−0.320
*p-value*	**<0.001**	**0.002**	0.123	**0.002**	**0.001**
Affective empathy	−0.233	−0.150	0.027	−0.297	−0.060
*p-value*	**0.004**	0.131	0.790	**0.002**	0.548
**PANSS total score**	0.206	0.180	0.233	−0.008	0.035
*p-value*	**0.027**	0.053	**0.012**	0.935	0.712
Positive	0.120	0.072	0.217	−0.007	−0.040
*p-value*	0.203	0.441	**0.019**	0.944	0.674
Negative	0.136	0.182	0.136	−0.130	0.077
*p-value*	0.146	0.051	0.147	0.163	0.413
General psychopathology	0.209	0.172	0.199	0.055	0.044
*p-value*	**0.025**	0.065	**0.032**	0.555	0.641
**Insight scale**	−0.022	−0.064	0.041	0.018	−0.045
*p-value*	0.818	0.494	0.660	0.848	0.635
**Calgary scale**	0.126	0.033	0.315	0.060	−0.147
*p-value*	0.181	0.726	**0.001**	0.523	0.116
**Age**	0.034	0.137	−0.127	0.048	0.033
*p-value*	0.680	0.091	0.118	0.559	0.685
**Chlorpromazine equivalent dose**	0.116	0.162	0.036	0.036	0.061
*p-value*	0.218	0.083	0.702	0.698	0.518^a^

a Values in bold indicate statistical significance (p < 0.05).

Moreover, no significant correlations were found between the ACSo scores (total or subscales) and insight, age, or chlorpromazine equivalent dose.

**Fig. 1:**
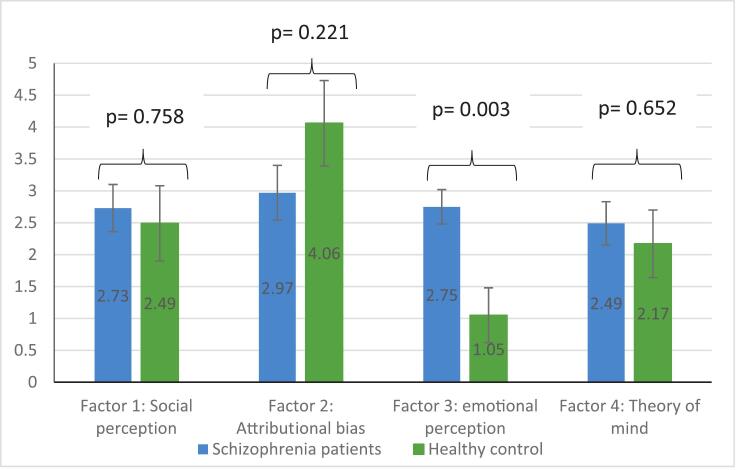
Mean values of the ACSo subscales by type of participants (schizophrenia patients vs. healthy controls) adjusted for age, sex, education level, family history of psychiatric illness, facial emotion recognition, theory of mind, empathy, and self-assessment scale of cognitive complaints.

Adjusted mean scores for ACSo subscales by group (schizophrenia patients vs. healthy controls) are shown in Fig. 1, controlling for age, sex, education level, family history of psychiatric illness, facial emotion recognition (TREF), theory of mind (TOM-15), empathy (QCAE), and cognitive complaints (SASCCS).

After adjusting for covariates, the emotional perception subscale differed significantly between groups, with patients with schizophrenia scoring higher than healthy controls (2.75 vs. 1.05, *p* = 0.003). No significant group differences were found for the remaining ACSo subscales: social perception, theory of mind, or attributional bias (all *p* > 0.05).

## 4 Discussion

This study represents the first translation and validation of the ACSo into Arabic among a sample of Lebanese patients with schizophrenia. The validated Arabic version demonstrated moderate internal consistency (Cronbach's alpha = 0.656), good construct validity, and adequate convergent validity, supporting its utility for assessing patients' self-perceived social cognitive impairment in schizophrenia.

The 4-factor structure identified in this study aligned with the original conceptual model, comprising emotional processing, theory of mind, social perception and knowledge, and attributional bias. These four dimensions accounted for 57.49 % of the total variance, slightly lower than the 64 % reported in the original French validation study with 89 patients (Graux et al., 2019). However, apart from the attributional bias factor, the three remaining factors identified differed in composition from the original version. These differences in factor structure imply cultural variations in the ACSo factor structure, which may be explained by differential item functioning across cultures. Individuals from different cultural backgrounds may have distinct perspectives on understanding and accepting the questions posed. For Arabic individuals with schizophrenia, responses may be influenced by collectivist values, family-centered relationships, and context-dependent social expectations, whereas French individuals may interpret the same questions differently due to more individualistic social norms (Soubra et al., 2024; Varnum et al., 2010). A study has demonstrated that differences in social orientation are responsible for cultural differences in cognitive style. More detailed comparisons have shown that, within a single culture, groups with different social orientations also exhibit differences in cognitive style (Varnum et al., 2010). Additionally, differences in factors between cultural groups may reflect true psychological differences, but they can also result from methodological, translation, or contextual factors, making cross-cultural comparisons open to multiple interpretations (Ember and Ember, 2009). Previous research has shown that other social cognition measures, such as the OSCARS, validated in 113 Arab patients with schizophrenia, also exhibited culturally influenced differences in factor structure, indicating the importance of using culturally adapted tools (Fekih-Romdhane et al., 2025). Another contributing factor is that this study's participants were chronic inpatients living in a close residential setting with limited social interactions outside this environment, which may have influenced their responses and potentially affected factor rotation in the analysis. The moderate correlation between factors and the total scale indicates that items are measuring the intended constructs consistently. However, since social cognitive factors inherently overlap, the specificity of individual items may be affected by this conceptual interdependence.

Regarding the correlation between factors and individual items, items designed to measure emotional processing, theory of mind, and social perception were positively correlated with their respective factors. However, item 12, which measures attributional bias, also correlated with the emotional processing factor. This finding is consistent with literature demonstrating significant conceptual and measurement-related overlap among social cognitive domains and that the boundaries between them are not clearly defined (Graux et al., 2019; Green et al., 2008). For instance, while recognizing emotions is undoubtedly a core component of emotional processing, it is sometimes considered to belong to the theory of mind (Green et al., 2008). Additionally, more than half of the patients included in this study were diagnosed with paranoid schizophrenia, which may explain the correlation between the attributional bias item and the emotional processing factor, as paranoid symptoms often involve both emotional reactivity and biased attributions (Bagrowska et al., 2025; Lam et al., 2021).

The positive correlation between the ACSo and SASCCS total score demonstrates concurrent validity, indicating that subjective social cognitive complaints align with general subjective cognitive complaints. This finding is consistent with those reported by Graux and colleagues (Graux et al., 2019). Similarly, another study found a positive correlation between the ACSo and SASCCS scales (*r* = 0.62) (Haddad et al., 2021).

The negative correlations observed between the ACSo total score and both the Facial Emotion Recognition Test (TREF) and neuropsychological assessments of theory of mind (TOM-15) demonstrated that higher levels of self-assessed social cognitive impairments were associated with poorer facial emotion recognition and theory of mind abilities. This finding aligns with existing literature indicating that deficits in these domains are common in individuals with schizophrenia and further validates the sensitivity of the ACSo to these impairments. However, no significant correlations were found between the emotional perception subscale and TREF, nor between the theory of mind subscale and TOM-15. This result is consistent with those reported by Graux and colleagues, who attributed these findings to the altered “social cognitive insight” in people with serious mental illness (Graux et al., 2019). Alternatively, the lack of correlation between objective assessments and the corresponding ACSo subscales may reflect the fact that the objective assessments focused on two specific aspects (facial emotion recognition and first- and second-order theory of mind) rather than the broader constructs assessed by the subscales (Graux et al., 2019).

Consistent with the results reported by Graux and colleagues (Graux et al., 2019), a negative association was found between ACSo and QCAE affective scores. In contrast to Graux et al., this study also observed a negative correlation between ACSo and the cognitive empathy component. This finding adds validity to the ACSo, demonstrating its ability to capture deficits in both empathy domains. Of note, a significant difference was found in cognitive empathy between patients and healthy controls; however, no difference was found in affective empathy. Healthy controls had higher cognitive empathy scores compared to the patient group. A Lebanese study among patients with schizophrenia and healthy controls found a significant difference in performance-based measures of empathy between the control and schizophrenia groups (Atoui et al., 2018). Another study among 35 patients with schizophrenia and healthy controls demonstrated a group difference in cognitive empathy but found no significant difference in emotional empathy (Berger et al., 2019).

Positive correlations were found between the ACSo total score and both the PANSS total score and PANSS general psychopathology, particularly the attributional bias subscale, indicating that as overall psychopathology and symptom severity increase, individuals tend to exhibit a higher level of self-assessed social cognitive impairments. The present study failed to demonstrate an association between ACSo and insight, supporting earlier findings of weak associations between subjective social complaints and insight (Haddad et al., 2021). No correlation was detected between the ACSo scale, subscales, and the total chlorpromazine equivalent dose. This result was expected because social cognition is not affected by the antipsychotic therapy targeting remission of clinical symptoms of schizophrenia (Javed and Charles, 2018).

In the current study, the ACSo showed moderate internal consistency, similar to the original work, where the four subscales (AB, SP, EP, and ToM) demonstrated moderate or good internal consistency (0.672, 0.647, 0.645, and 0.675, respectively) (Graux et al., 2019). A Lebanese study reported a higher Cronbach's alpha value of 0.767 for the ACSo (Haddad et al., 2021); however, it is worth noting that the Lebanese study used the 20-item version rather than the 12-item version employed here (Haddad et al., 2021). The original ACSo development involved pilot testing with a 20-item questionnaire organized into four groups, followed by a shorter 12-item version following evaluation of its psychometric properties (Graux et al., 2019). In a multi-domain scale with a valid multifactorial structure, lower internal consistency is expected, as the items measure distinct constructs (Edelsbrunner et al., 2025). Cronbach's alpha is also sensitive to the number of items in the scale and generally tends to underestimate internal consistency reliability. In addition, the ACSo is a composite scale assessing several processes of social cognition, which may explain the moderate Cronbach's alpha values. Composite reliability values of 0.60 to 0.70 are acceptable in exploratory research, while in more advanced stages of research, values between 0.70 and 0.90 can be regarded as satisfactory (Nunnally, 1967). Sample size may also have influenced the Cronbach's alpha obtained in this study.

### 4.1 Limitation

This study has several limitations. The sample consisted of chronically hospitalized patients whose cognitive function might be severely impaired, raising the possibility of selection bias. Moreover, the study results cannot be generalized to broader populations due to the small sample size and the fact that patients were recruited from a single site. Due to the relatively small sample size, it was not feasible to split the sample to perform a confirmatory factor analysis (CFA) following the EFA, which limits the ability to fully confirm the factor structure of the ACSo in our sample. Additionally, the healthy control group was recruited from hospital employees, which could not adequately reflect the characteristics of the general population. Test-retest reliability of the scale's temporal stability and reproducibility was not assessed. Finally, recall bias might have occurred, as participants may have been unable to give accurate details during the face-to-face interview.

## 5 Conclusion

The Arabic version of the ACSo demonstrated appropriate psychometric properties with moderate internal consistency, good construct validity, and adequate concurrent validity, making it a valuable tool for assessing subjective social cognitive complaints in Lebanese patients with schizophrenia. This validated Arabic ACSo can serve as an accessible instrument that can be readily included in research studies and clinical settings to complement psychosocial rehabilitation assessments with subjective evaluation of social cognition, ultimately supporting patients in achieving better social integration.

The following are the supplementary data related to this article.

Table S1 Pearson correlation between the ACSo items among patients with schizophrenia Table S1

## CRediT authorship contribution statement

**Chadia Haddad:** Writing – original draft, Methodology, Investigation, Conceptualization. **Samah Al Aswad:** Writing – original draft, Project administration, Methodology. **Hala Sacre:** Writing – review & editing, Validation. **Francois Kazour:** Writing – review & editing, Validation, Resources. **Helen Clery:** Writing – review & editing, Resources. **Jérôme Graux:** Writing – review & editing, Resources. **Sara Al Kadamani:** Writing – review & editing, Project administration, Methodology. **Adella Ibrahim:** Writing – review & editing, Project administration, Methodology. **Anthony Rizk:** Writing – review & editing, Project administration, Methodology. **Pascale Salameh:** Writing – review & editing, Validation, Supervision. **Benjamin Calvet:** Writing – review & editing, Validation, Supervision, Conceptualization.

## Consent for publication

Not applicable.

## Ethics approval and consent to participate

The Ethics and Research Committee at the Psychiatric Hospital of the Cross approved this study (HPC-004-08-23) in compliance with the Hospital's Regulatory Research Protocol. The purpose and requirement of the study were explained to each participant. Consent was obtained in writing on the informed consent form.

## Funding

None.

## Declaration of competing interest

The authors declare that they have no competing interests.

## Data Availability

The datasets supporting the conclusions of this article are available from the corresponding author on reasonable request.
