# Supplementary material for: Validation of the Arabic version of the self-assessment of social cognitive impairments (ACSo) tool among a sample of patients with schizophrenia
Source: Schizophr Res Cogn. 2026 Jan 6;44:100417. doi: 10.1016/j.scog.2025.100417 (PMC12811678; doi:10.1016/j.scog.2025.100417)
Supplement: Table S1 — Pearson correlation between the ACSo items among patients with schizophrenia [file mmc1.docx]

**Supplementary file**

| **Table S1: Pearson correlation between the ACSo items among patients with schizophrenia** | | | | | |
| --- | --- | --- | --- | --- | --- |
|  | **Total ACSo** | **Factor 1:**  **Social perception** | **Factor 2: Attributional bias** | **Factor 3: Emotional perception** | **Factor 4:**  **Theory of mind** |
| ACSO Item 1 | 0.588 | 0.773 | 0.042 | 0.289 | 0.361 |
| *p-value* | **<0.001** | **<0.001** | 0.654 | **0.002** | **<0.001** |
| ACSO Item 2 | 0.616 | 0.819 | 0.091 | 0.310 | 0.302 |
| *p-value* | **<0.001** | **<0.001** | 0.332 | **0.001** | **0.001** |
| ACSO Item 3 | 0.446 | 0.315 | 0.005 | 0.167 | 0.675 |
| *p-value* | **<0.001** | **0.001** | 0.962 | 0.074 | **<0.001** |
| ACSO Item 4 | 0.403 | 0.009 | 0.781 | 0.088 | 0.069 |
| *p-value* | **<0.001** | 0.923 | <0.**001** | 0.348 | 0.463 |
| ACSO Item 5 | 0.251 | 0.070 | 0.463 | 0.104 | -0.063 |
| *p-value* | **0.007** | 0.458 | **<0.001** | 0.267 | 0.505 |
| ACSO Item 6 | 0.416 | 0.183 | -0.043 | 0.240 | 0.740 |
| *p-value* | **<0.001** | **0.049** | 0.650 | **0.010** | **<0.001** |
| ACSO Item 7 | 0.284 | 0.041 | 0.651 | -0.144 | 0.048 |
| *p-value* | **0.002** | 0.664 | **<0.001** | 0.123 | 0.607 |
| ACSO Item 8 | 0.508 | 0.237 | 0.053 | 0.818 | 0.324 |
| *p-value* | **<0.001** | **0.010** | 0.574 | **<0.001** | **<0.001** |
| ACSO Item 9 | 0.492 | 0.772 | -0.063 | 0.189 | 0.300 |
| *p-value* | **<0.001** | **<0.001** | 0.501 | **0.042** | **0.001** |
| ACSO Item 10 | 0.623 | 0.394 | 0.179 | 0.375 | 0.661 |
| *p-value* | **<0.001** | **<0.001** | 0.055 | **<0.001** | **<0.001** |
| ACSO Item 11 | 0.508 | 0.298 | 0.075 | 0.773 | 0.259 |
| *p-value* | **<0.001** | **0.001** | 0.426 | **<0.001** | **0.005** |
| ACSO Item 12 | 0.365 | -0.016 | 0.659 | 0.199 | 0.041 |
| *p-value* | **<0.001** | 0.866 | **<0.001** | **0.033** | 0.663 |
| **Total ACSo** |  | 0.716 | 0.506 | 0.638 | 0.687 |
| *p-value* |  | **<0.001** | **<0.001** | **<0.001** | **<0.001** |
| **Factor 1:**  **Social perception** | 0.716 |  | 0.030 | 0.334 | 0.408 |
| *p-value* | **<0.001** |  | 0.749 | **<0.001** | **<0.001** |
| **Factor 2: Attributional bias** | 0.506 | 0.030 |  | 0.079 | 0.051 |
| *p-value* | **<0.001** | 0.749 |  | 0.398 | 0.584 |
| **Factor 3: Emotional perception** | 0.638 | 0.334 | 0.079 |  | 0.368 |
| *p-value* | **<0.001** | **<0.001** | 0.398 |  | **<0.001** |
| **Factor 4:**  **Theory of mind** | 0.687 | 0.408 | 0.051 | 0.368 |  |
| *p-value* | **<0.001** | **<0.001** | 0.584 | **<0.001** |  |
